# Quantitative Risk Assessment Model of Human Salmonellosis Resulting from Consumption of Broiler Chicken

**DOI:** 10.3390/diseases7010019

**Published:** 2019-02-07

**Authors:** Luma Akil, H. Anwar Ahmad

**Affiliations:** 1 Department of Behavioral and Environmental Health, School of Public Health, Jackson State University, Jackson, MS 39211, USA; luma.akil@jsums.edu; 2 Department of Biology/Environmental Science, College of Science, Engineering and Technology, Jackson State University, Jackson, MS 39217, USA

**Keywords:** *Salmonella*, broiler chicken, risk assessment, farm-to-fork, food safety

## Abstract

(1) Background: *Salmonella* infections are a major cause of illnesses in the United States. Each year around 450 people die from the disease and more than 23,000 people are hospitalized. *Salmonella* outbreaks are commonly associated with eggs, meat and poultry. In this study, a quantitative risk assessment model (QRAM) was developed to determine *Salmonella* infections in broiler chicken. (2) Methods: Data of positive *Salmonella* infections were obtained from the United States Department of Agriculture (USDA) and the Centers for Disease Control and Prevention (CDC) Foodborne Disease Outbreak Surveillance System, in addition to published literature. The Decision Tools @RISK add-in software was used for various analyses and to develop the QRAM. The farm-to-fork pathway was modeled as a series of unit operations and associated pathogen events that included initial contamination at the broiler house (node 1), contamination at the slaughter house (node 2), contamination at retail (node 3), cross-contamination during serving and cooking (node 4), and finally the dose–response model after consumption. (3) Results: QRAM of *Salmonella* infections from broiler meat showed highest contribution of infection from the retail node (33.5%). (4) Conclusions: This QRAM that predicts the risk of *Salmonella* infections could be used as a guiding tool to manage the *Salmonella* control programs

## 1. Introduction

*Salmonella* is a critical foodborne pathogen worldwide with an estimated 1.2 million illnesses, 23,000 hospitalizations, and 450 deaths in the United States of America (USA) every year [1]. Non-typhoidal *Salmonella* infections are the most commonly reported bacteriological agent of human foodborne diseases in the USA. It was estimated to contribute 11% of foodborne human illnesses, 35% of hospitalizations, and 28% of deaths attributable to infections by known foodborne pathogens, thereby ranking highest among all bacterial foodborne pathogens with approximately 44% of confirmed foodborne bacterial infections [1,2,3]. Prevalence of several factors including geographical location, climatic variability, farming practice, socioeconomic factors and consumer habits contributes to differences in *Salmonella* infections and incidences [4,5].

Raw poultry and meat products consumption remain the principal source of *Salmonella* in many countries. *Salmonella* has been reported in a variety of chicken, turkey and other meat products, in addition to fresh produce such as lettuce and sprouts [6]. Studies have reported that poultry is found to be associated with 25% of outbreaks, illnesses, and hospitalizations caused by a confirmed foodborne pathogen [7,8]. The main reservoir of non-typhoidal *Salmonella* is the intestinal tract of animals, which will easily lead to contamination of diverse food products. *Salmonella* is carried by different animals and may contaminate fresh water by direct or indirect contact, which may lead to contamination of fresh produce as well. Poultry is frequently colonized with *Salmonella* without detectable symptoms. As a result, it was suggested that poultry is the main human health risk factor, as it allows the bacteria to easily transmit from table eggs and poultry meat to humans [9].

*Salmonella* serotypes associated with human infections are able to persist along the food chain (during the primary production, slaughter operations, equipment, meat handlers, and retail). As live birds are processed, the existing bacterium is introduced to the poultry production system, and each stage of processing is a potential point for the environment for *Salmonella* contamination. *Salmonella* could, therefore, spread from carcass to carcass along the processing stages [9]. During any of the steps in the farm-to-consumer continuum, further microbial contamination can occur from a variety of sources, such as environmental, animal or human, including risk of pathogenic microorganisms [10,11]. The efficient transmission and the rapid spread are of public health relevance. A greater understanding of salmonellosis and the source that contributes to the high rates of the disease is very critical.

In food safety, a quantitative risk assessment model (QRAM) describing the risk pathway usually begins with determination of the hazard on the farm, and ends with the dose received by the consumer or the number of human illness cases [12]. Risk assessment provides estimates on the probability of disease occurrence based on four steps: hazard identification, exposure assessment, hazard characterization (dose–response relationship), and risk characterization. There is, therefore, a strong need to provide data on the frequency and level of *Salmonella* contamination in meat and meat products. The QRAM aims to describe the dynamics of bacteria in a food chain and its concentration per unit of a given food item. This variation is dependent on the variable describing the number of bacteria at a previous stage or node and associated process parameters, like temperature, time, and acidity. In each module, one or more of the basic microbiological processes and processing stages of inactivation, growth, partitioning, mixing, removal and cross-contamination are modeled. These processes are defined by functional relations, having the process parameters and the concentrations of the bacteria at a previous module as arguments of the functions. The final model is obtained by linking the nodes, passing information from one node to the next [12,13].

Predictive food microbiology is a rapidly developing area of food microbiology. It allows estimating the contamination levels of pathogens at different stages to ensure safety and quality of food product. QRAM is widely used in literature to estimate the probability of *Salmonella* infections at different stages of food production. In this research, a general framework to understand the contributing sources of *Salmonella* during the broiler production process is explored.

Objectives: Risk assessment is widely used by governmental and regulatory agencies worldwide to protect public health from exposure to a myriad of contaminants through numerous routes of exposure. The objective of this study is to determine human risk of *Salmonella* due to broiler consumption using QRAM from the farm-to-fork infection pathway.

## 2. Materials and Methods

Quantitative risk assessment uses probability distributions to model the variability and uncertainty of important risk factors, such as time, temperature and pathogen density. The QRAM is simulated using a spreadsheet add-in program that randomly samples the probability distributions and uses the random numbers generated to perform calculations and generate output distributions [13]. The QRAM forecasts the risk using Monte Carlo simulation. The basic idea behind the Monte Carlo simulation procedure is that the simulation is iteratively refined during a number of iterations, giving feasible representations of a real-world situation. At each iteration, numbers are drawn from the probability distributions representing the variability of the given process. The number of iterations is preset, or dependent on the convergence behavior of the simulation. The results of iterations are stored. At the end, statistical methods may be employed in order to extract summary data from the results [13,14,15].

In this study, a quantitative risk assessment for *Salmonella* in broiler chicken was constructed in a Microsoft Excel (Microsoft, Redmond, WA) spreadsheet and was simulated using the Decision Tools @Risk (version 7.5.1, Palisade, Newfield, NY), which is an add-in for Microsoft Excel that performs risk analysis on any spreadsheet model by using Monte Carlo simulation.

**Figure 1:**
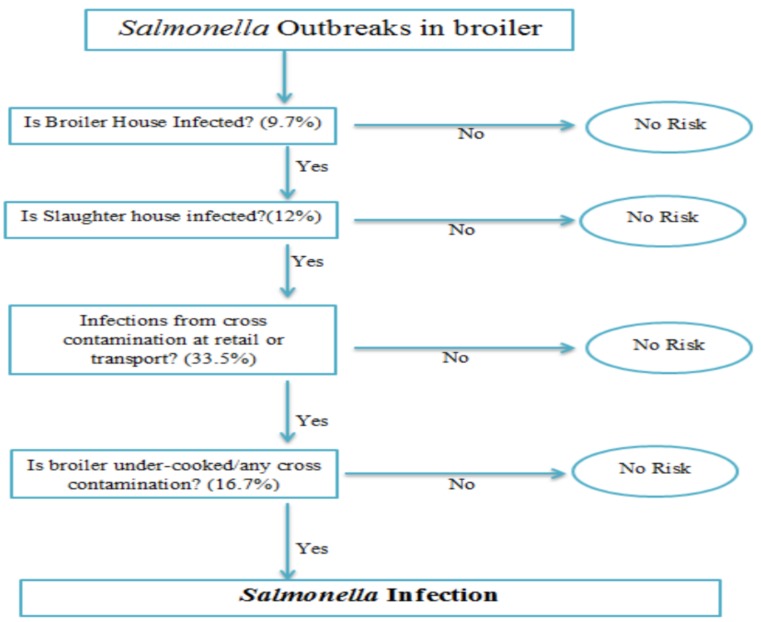
Probability framework of *Salmonella* infections at different nodes using the farm-to-fork pathway.

The farm-to-fork pathway was modeled as a series of unit operations and associated pathogen events that included initial contamination at the broiler house (node 1), contamination at the slaughter house (node 2), contamination at retail (node 3), cross-contamination during serving and cooking (node 4), and finally the dose–response model after consumption (Figure 1).

### 2.1. Risk Assessment Model

#### 2.1.1. Node One: Broiler House

The United States Department of Agriculture Food Safety and Inspection Service (USDA FSIS) uses data from its regulatory testing programs to monitor the effectiveness of its Pathogen Reduction/Hazard Analysis and Critical Control Point Systems Final Rule, and to assess process control in individual establishments [16,17]. The U.S. Department of Health and Human Services has incorporated the target of 11.4 cases of salmonellosis/100,000 persons into the Healthy People 2020 objectives aimed at a 25 percent reduction in human illnesses (HealthyPeople.gov, 2018), which FSIS recognizes as appropriate guidance for the agency’s strategic planning to strengthen public health protection.

Data of percent positive *Salmonella* test were obtained from the USDA FSIS “Progress Report on *Salmonella* and *Campylobacter* Testing of Raw Meat and Poultry Products, 1998–2011” [16]. The average percent of positive *Salmonella* in broiler chicken from 2003 to 2011 is given in Table 1.

The data were graphed in Excel and the distributions for the node was defined using @RISK define distribution function. The gamma distribution was the appropriate selection for this type of data based on the slope; and related parameters, alpha (3.284) and beta (3.45), were generated using Excel. Monte Carlo simulation with 1000 iterations using alpha and beta values was selected. During the simulation, @Risk randomly samples values across the full range of the curve and recalculates the spreadsheet using random values sampled from the input distribution functions and record the output values to determine the risk for the first node. For each iteration, a new randomly generated value is placed in the Excel cell. When the simulation is completed, the results are shown in the @Risk’s results window. 

#### 2.1.2. Node Two: Slaughter House 

The second node of the risk assessment model simulated the data of positive *Salmonella* testing at the slaughter house. Data of positive *Salmonella* at the slaughter house were obtained from “FSIS Risk Assessment for Guiding, Public Health-Based Poultry, Slaughter Inspection”, USDA FSIS 2012 and 2008 reports [17].

The PERT distribution (also called three-point estimation) was defined and selected for node two as the appropriate distribution with minimum, most likely and maximum values of 2.2, 8.49 and 35.5, respectively, which were generated using Excel. Monte Carlo simulation with 1000 iterations using minimum, most likely and maximum values was selected. The simulation generated the risk for the second node.

#### 2.1.3. Node Three: Retail 

Reviews of the scientific literature indicated that the incidence of *Salmonella* contamination of broiler chickens is variable among studies because of differences in chicken production and processing practices [13] as well as differences in sampling and detection methods. Several studies had examined *Salmonella* outbreaks from retail sources [7,13,18,19]. Data of these studies were collected and used to simulate and generate the risk of *Salmonella* contamination at the retail level. A minimum value of 1.57, a median value of 30.57, and a maximum value of 77.14 of positive *Salmonella* were calculated based on the collected data, and used to define the input settings for the PERT distribution for the extent of *Salmonella* contamination of broiler chickens at the retail level. The data were simulated and 1000 iterations were generated to determine the risk for the third node.

#### 2.1.4. Node Four: Cooking and Mishandling 

The fourth node simulated thermal inactivation of *Salmonella* during cooking and cross-contamination of cooked chickens with *Salmonella* during serving. Thermal inactivation of *Salmonella* depends on a number of risk factors, such as time, temperature, product shape and size, strain of *Salmonella*, methods of cooking, and physiological states of *Salmonella*. In this node, incidence refers to the percentage of chickens that were mishandled by consumers, for example cutting of cooked chicken with utensils used to prepare raw chicken.

A minimum value of 0.6, a median value of 14.57, and a maximum value of 41.7, obtained from several literature articles of positive *Salmonella* [7,13,18,19,20,21], were used to define the input settings for the PERT distribution for the extent of *Salmonella* contamination of broiler chickens for consumers. The data were simulated and 1000 iterations were generated to determine the risk for the fourth node.

Finally, all nodes were combined together to perform a concluding simulation of 10,000 iteration using @RISK software to determine the overall risk of *salmonella* infection from all stages of farm-to-fork pathway.

**Figure 2:**
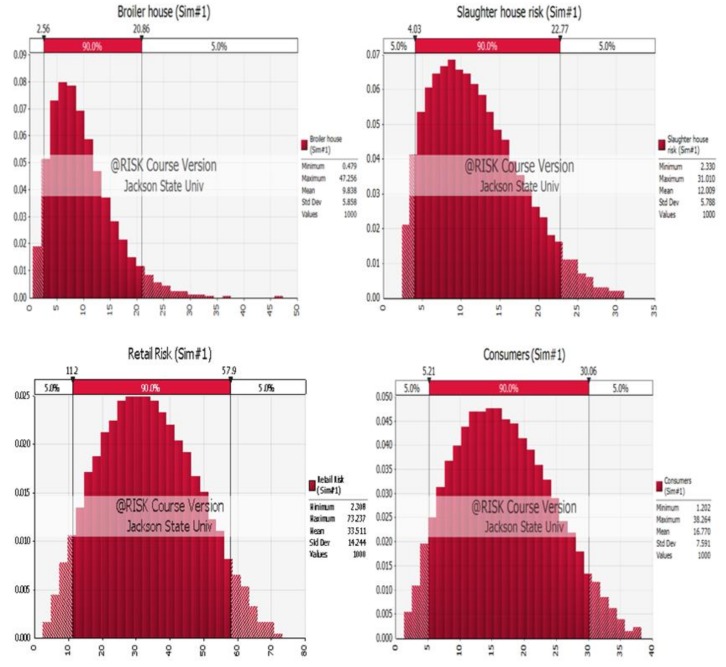
Simulation models for the four nodes of the Quantitative Risk Assessment Model (QRAM): broiler house, slaughter house, retail and consumers.

Simulation models of the four nodes are shown in Figure 2.

### 2.2. Consumption—Dose–Response Model

Dose–response data for nontyphoid *Salmonella* in humans are limited to a large feeding trial with healthy men [22]. Data from the study have been extensively modeled and indicate differences in virulence among strains of *Salmonella*. 

Data for dose–response model were obtained from a study by Teunis [23], and The Food and Agriculture Organization (FAO) dose–response model for *Salmonella* [24]. The concentration, number of persons who are ill and number of exposed were obtained and used in Probit regression analysis. Software package SPSS 21 was used to carry out the regression analysis using the number of ill as response, total exposed as total observation and log dose mean as a covariate. Infectious concentration 50 was calculated by finding the concentration at a probability of 50% illness, using GraphPad software package. 

## 3. Results

Detailed results of risk assessment model are summarized in Table 2.

A QRAM was carried out, based on the understanding of production processes of broiler chicken, to determine the risk of *Salmonella* infections from the farm-to-fork pathway. The QRAM was modeled as a series of unit operations and associated pathogen events that included initial contamination at the broiler house (node 1), contamination at the slaughter house (node 2), contamination at the retail level (node 3), and cross-contamination during serving and cooking (node 4).

Node 1: data of *Salmonella* infections at the broiler house simulation using @RISK software randomly assigned an output of risk for this node creating minimum, maximum, and mean values of 0.55, 40.23, and 9.79, respectively.

Node 2: positive *Salmonella* rates at the slaughter house simulation was carried out and a random risk output for this node was generated, which were 2.33, 30.64, and 11.94, for minimum, maximum and mean values, respectively. 

Node 3: *Salmonella* infection at the retail level, including data of transport, cross-contamination at retail, and contamination related to packaging, was simulated and the random risk generated for this node as a result of minimum, maximum and means values of 1.57, 74.16 and 33.5, respectively.

Node 4: data from literature review of *Salmonella* infection at the consumer level included cooking to improper temperature, cross-contamination with raw chicken and improper hygiene practices simulation random risk generated for this node as a result of minimum, maximum and means values of 1.34, 39.26, and 16.76, respectively.

Collectively, the outputs of all nodes were combined for further risk simulation with 10,000 iterations. This resulted in random risk of 1.85, 45.33, and 19.92 for minimum, maximum and mean values, corresponding to the best, worst and average scenario cases of *Salmonella* infections in broiler chicken, respectively. 

Results showed that infection at retail caused the highest risk of *Salmonella* with a mean of 33.5%. Based on broiler production and consumption, from farm to fork, around 53 out of 100,000 broiler chickens will be infected with *Salmonella*. Contaminated broiler chicken will cause an estimated 19 cases in 100,000 of *Salmonella* infections in human. Mississippi contributes to ten percent of broiler chickens produced in the USA, resulting in 5 infected broiler chickens in 100,000 and causing 2 cases in 100,000 of *Salmonella* infections in human.

### Dose Response of Salmonella 

Not all individuals in a population are equally susceptible to *Salmonella* infections. In order for the human to be infected and show symptoms of salmonellosis, consumption of infectious dose of *Salmonella* in broiler chicken is required.

**Figure 3:**
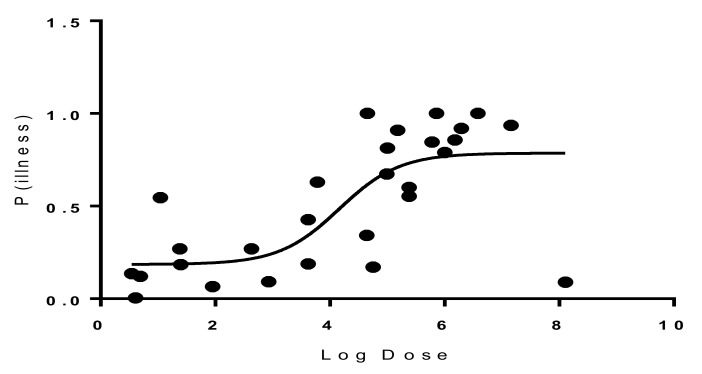
Dose–response graph for *Salmonella enterica* serovar Enteritidis.

**Figure 4:**
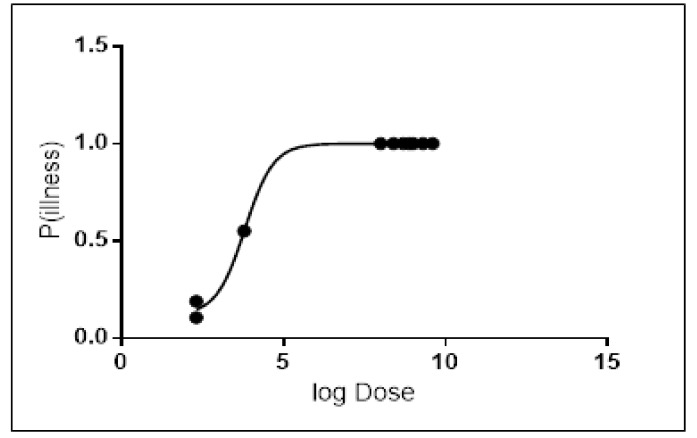
Dose–response graph for *Salmonella enterica* serovar Typhimurium.

Probit regression analysis was carried out to determine the Infectious dose-50 (ID-50) for the most common serotypes of *Salmonella* resulting from broiler chicken consumption. Results showed that the ID-50 values of *Salmonella enterica* serovar Enteritidis were 1.46 × 10^4^ (Figure 3), and 6.4 × 10^3^ for *Salmonella enterica* serovar Typhimurium (Figure 4). On an average, consumption of at least 1.46 × 10^4^ CFU/g for *Salmonella enterica* serovar Enteritidis or 6.4 × 10^3^ CFU/g for *Salmonella enterica* serovar Typhimurium is required to develop infection in 50% of the population. 

## 4. Discussion

Our QRAM of *Salmonella* infections from the farm-to-fork pathway determined the highest risk of *Salmonella* infection at the retail level with a mean of 33.5%. The World Health Organization/ Food and Agriculture Organization (WHO/FAO) published an international risk assessment on *Salmonella* in broiler production. One of their key findings was that a reduction in the prevalence of *Salmonella*-contaminated chicken was correlated with a reduction in the risk of human illness. For instance, a 50% reduction in the prevalence of contaminated poultry (20% to 10%) produced a 50% reduction in the expected risk of illness per serving. Similarly, a large reduction in prevalence (20% to 0.05%) would produce a 99.75% reduction in the expected risk of illness [25]. 

In the current study, positive *Salmonella* infections at the broiler house (node 1) were shown to contribute around 10% of *Salmonella* risk. Newly hatched broilers are highly susceptible to *Salmonella* colonization, likely due to the composition of their intestinal microbiota [26,27]. Over the last 50 years in Southern USA, grow-out broilers in intensive production systems have been housed on deep litter on the floor. In these economy-of-scale production systems, the birds are placed into grow-out houses within a day after hatch, directly on litter. Therefore, if *Salmonella* is present in the litter, the birds are exposed at a time when they are highly susceptible. In fact, the presence of *Salmonella* in the grow-out house, specifically in the litter, prior to placement of a new flock and contamination of the previous flock reared in the house, has shown to be precursors of higher *Salmonella* frequencies in the new flock at later stages of the production continuum [26]. *Salmonella* may persist in dry livestock buildings for many months. A high standard of disinfection is necessary to avoid infection of poultry placed in a previously infected house [27].

The risk assessment model showed that risk at the slaughter house is estimated to contribute about 12% of *Salmonella* infections. Other studies also developed a QRAM of *Salmonella* from slaughtered broiler flocks to consumers [20]. Their model estimated that approximately 0.21% of domestically produced broiler meat mass was contaminated with *Salmonella*. They also suggested that the effect of eliminating breeder flocks from production which have tested positive for *Salmonella* and heat-treating the meat of detected positive broiler flocks would result in 1.0–2.5 less reported human cases compared to the expected number of cases [20].

Further, the current risk assessment model showed that risk of *Salmonella* infections caused from a retail source had the highest contribution of infections (33%). Furthermore, a study examined *Campylobacter* and *Salmonella* in 300 raw samples (whole chicken, chicken breast with skin or chicken pieces) that were purchased on a monthly basis for seven months. They reported that *Campylobacter* and *Salmonella* were isolated from 68% and 29% of retail chicken, respectively. They found that *Salmonella* was absent from external packaging but was isolated from 11% of whole packaging [28]. This study demonstrated that both chicken and chicken packaging are vehicles for potential cross-contamination of *Campylobacter* and *Salmonella*. In addition, it was reported that broiler chickens at production were contaminated at a rate of 2.49%, and increased during transportation to the processing plants (3.95%), processing (51.32%) and retail (77.14%). It was suggested that the lower infection rates during production may be due to voluntary control measures to reduce microbial infection in the broiler population, which translates to an increase in profits. However, higher *Salmonella* infection rates during processing and retail were due to cross-contamination from processing procedures, and handling during these stages [18].

A risk reduction up to 17% was observed in the current risk assessment model for the consumer node. Although the presence of *Salmonella* in poultry is relatively common, poultry can be safely consumed when it is cooked at a safe internal endpoint temperature [29]. Reduction in concentration of *Salmonella* on chicken at retail, and washing cutting boards (or utensils) and hands after handling raw chicken and proper cooking at consumers’ homes can result in a marked reduction in the predicted probability of illness. The cooking times required to reach a set endpoint temperature differed considerably. Cooking times differed between individual poultry in the same class and cooked in the same oven. Factors most likely contributing to the variability both between and within individual poultry include differences in shapes, proportions of white and dark meat and fat distribution. Furthermore, the position of the poultry in the cooking pans could have impacted the rate of cooking in various regions of the poultry. The USDA recommended a cooking temperature of 74 °C for chicken. A study found that an endpoint temperature of 74 °C, with a hold time of less than 10 min for both chicken and turkey, could achieve a 7-log reduction of *Salmonella* [29]. Another study by Oscar found that incidence of *Salmonella* contamination was 30% at retail and it changed to 0.16% after cooking and then to 4% at consumption. Their model predicted 0.44 cases of salmonellosis per 100,000 consumers, which was consistent with recent epidemiological data that indicate a rate of 0.66–0.88 cases of salmonellosis per 100,000 consumers of chicken [13].

*Salmonella* rates have significantly declined in farms due to technological advances and improved preventive strategies in addition to hygiene and control measures at various production levels. Some of these preventive strategies include feed and drinking water acidification with organic acids, modification of nutrients and ingredients of bird feed to reduce their susceptibility to *Salmonella* infections. Additionally, use of feed additives, such as antibiotics, prebiotics, probiotics, and synbiotics, modifies the intestinal microflora, but may not be the best control measure of infections [30]. 

As a result, this risk assessment model can be used as a tool to organize management control programs to enhance awareness of the critical points, where *Salmonella* can be controlled the most by the food handlers at retail and private kitchens. Training and educational programs to enhance such practices should be encouraged. The outcome of the current model emphasizes that food safety is not one party’s responsibility; rather, it is a shared responsibility among all stakeholders in charge of handling the broiler chicken.

Not every exposure to a pathogen in food will result in infection or illness in humans, and not all individuals in a given population are equally susceptible to all pathogens. Therefore, the risk of food borne disease is a combination of the likelihood of exposure to a pathogen in a food, the likelihood that exposure will result in infection or intoxication and subsequently illness and the severity of the illness. On a population basis, a calculation of risk can predict the expected number of specific illnesses or deaths per 100,000 population per year attributable to the pathogen/food in question, or risk can be defined as the probability of a specific adverse outcome per exposure to the food [31]. The susceptibility of a person plays a major role in acquiring *Salmonella* infection. The calculated risk of infection is higher and more potent for children under 5 years old, infants who are not breastfed, adults over 65 years old, and people with weakened immune systems as they are most likely to have severe infections. In addition, certain medications, such as treatment of hyperacidity in stomach, can increase the risk of *Salmonella* infection as well [1]. 

### Dose Response of Salmonella 

In this study, a Probit regression analysis was constructed to determine the Infectious dose-50 (ID-50) for *Salmonella enterica* serovar Enteritidis and *Salmonella enterica* serovar Typhimurium. Results of this analysis showed that in *Salmonella enterica* serovar Enteritidis, ID-50 values were 1.46 × 10^4^ and 6.4 × 10^3^ for *Salmonella enterica* serovar Typhimurium, respectively. Our results were consistent with results of other studies that determined the dose response for salmonellosis in broiler chicken [13,24,28]. 

Other studies created separate dose–response models for infection and illness given infection using a multi-level statistical framework [23]. Those models incorporated serotype and susceptibility as categorical covariates, and adjusted for heterogeneity in exposure. Their results indicated that both the risk of infection and the risk of illness given infection increase with dose. The dose–response model incorporating data from all outbreaks had an infection ID-50 of 7 CFUs’ and illness ID-50 of 36 CFUs. However, for serotypes other than *Salmonella enterica* serovar Enteritidis and *Salmonella enterica* serovar Typhimurium, literature indicated that a minor proportion of individuals exposed will not fall ill even at high doses. The dose–response relations indicate that outbreaks are associated with higher doses making it more likely to have a higher attack rate. In healthy humans, the infectious dose is generally 10^6^ to 10^8^, but lower bacterial counts can cause disease in certain conditions, as well as in infants and the elderly. Although uncommon, life-threatening invasive infections with bacteremia (5%–10% of infected persons) and/or other extra-intestinal infections may occur, affecting especially the risk groups (infants, young children, older people and immunocompromised patients) [9]. The dose–response model based upon the observed outbreak data provides an estimate for the probability of illness that is based on real-world data. The outbreak model offers the best alternative for estimating the probability of illness upon ingestion of a dose of *Salmonella*.

Minimizing the risk of disease transmission is a major concern for governments and health professional organizations. Understanding the contributing source to the risk of disease will allow prevention and control measures to eliminate the disease. 

## 5. Conclusions

This *Salmonella* risk assessment provides information that should be useful in determining the impact intervention strategies may have on reducing cases of salmonellosis from contaminated broiler. Risk assessment, along with risk management and risk communication, is one of the components of risk analysis, which can be defined as an overall strategy for addressing risk. In this study, a farm-to-fork risk assessment determined that the highest rates of infections are from the retail level. However, proper hygiene and safe handling and cooking practices will reduce the rates of infections. The QRAM model that predict the risk of *Salmonella* infections could be used as a guiding tool to manage the disease control programs.

## Figures and Tables

**Table 1:** Percent positive *Salmonella* in broiler chicken during 2003–2011.

Year	Positive *Salmonella* %
2003	11.2
2004	13.5
2005	16.3
2006	11.4
2007	8.5
2008	7.3
2009	7.2
2010	6.7
2011	6.5

Data Source: The United States Department of Agriculture Food Safety and Inspection Service (USDA FSIS).

**Table 2:** Results of risk assessment model for *Salmonella* in broiler chicken.

Name	Risk-Broiler House	Risk-Slaughter House	Risk-Retail	Risk-Consumer	Final Risk
Description	GammaRisk	RiskPert	RiskPert	RiskPert	RiskPert
Minimum	0.55332	2.332349	1.574895	1.342847	1.856516
Maximum	40.23986	30.64444	74.1679	39.25882	45.33582
Mean	9.796366	11.94164	33.51049	16.76949	19.92174
Standard Deviation	5.804362	5.724642	14.24967	7.593811	8.308862
Variance	33.69061	32.77153	203.053	57.66596	69.03718

## References

[1] Centers for Disease Control and Prevention (CDC) Foodborne Illnesses and Germs. https://www.cdc.gov/foodsafety/foodborne-germs.html.

[2] Scallan E., Hoekstra R.M., Angulo F.J., Tauxe R.V., Widdowson M.A., Roy S.L., Jones J.L., Griffin P.M. (2011). Foodborne illness acquired in the United States--major pathogens. Emerg. Infect. Dis..

[3] Brichta-Harhay D.M., Arthur T.M., Bosilevac J.M., Kalchayanand N., Shackelford S.D., Wheeler T.L., Koohmaraie M., Koohmaraie M. (2011). Diversity of Multidrug-Resistant *Salmonella enterica* Strains Associated with Cattle at Harvest in the United States. Appl Environ. Microbiol..

[4] Akil L., Ahmad H.A., Reddy R.S. (2014). Effects of climate change on Salmonella infections. Foodborne Pathog. Dis..

[5] Akil L., Ahmad H.A. (2016). Salmonella infections modelling in Mississippi using neural network and geographical information system (GIS). BMJ Open.

[6] Rajan K., Shi Z., Ricke S.C. (2017). Current aspects of Salmonella contamination in the US poultry production chain and the potential application of risk strategies in understanding emerging hazards. Crit. Rev. Microbiol..

[7] Smadi H., Sargeant J.M. (2013). Quantitative risk assessment of human salmonellosis in Canadian broiler chicken breast from retail to consumption. Risk Analysis.

[8] Chai S.J., Cole D., Nisler A., Mahon B.E. (2017). Poultry: The most common food in outbreaks with known pathogens, United States, 1998-2012. J. Epidemiol. Infect..

[9] Antunes P., Mourão J., Campos J., Peixe L. (2016). Salmonellosis: The role of poultry meat. Clin. Microbiol. Infec..

[10] Mead P.S., Slutsker L., Dietz V., McCaig L.F., Bresee J.S., Shapiro C., Griffin P.M., Tauxe R.V. (1999). Food-related illness and death in the United States. Emerg. Infect. Dis..

[11] Denis G., Maki M.D. (2009). Coming to Grips with Foodborne Infection—Peanut Butter, Peppers, and Nationwide Salmonella Outbreaks. N. Engl. J. Med..

[12] Smid J.H., Verloo D., Barker G.C., Havelaar A. (2010). Strengths and weaknesses of Monte Carlo simulation models and Bayesian belief networks in microbial risk assessment. Int. J. Food Microbiol..

[13] Oscar T.P. (2004). A quantitative risk assessment model for Salmonella and whole chickens. Int. J. Food Microbiol..

[14] Qin L., Yang S.X., Meng M.Q.H. (2007). Mathematical Model with Degree of Risk for Salmonella Infections. Systems, Man and Cybernetics. ISIC.

[15] Sharma C.S., Ates A., Joseph P., Nannapaneni R., Kiss A. (2013). Reduction of Salmonella in skinless chicken breast fillets by lauric arginate surface application. Poult. Sci..

[16] U.S. Department of Agriculture Progress Report on Salmonella and Campylobacter Testing of Raw Meat and Poultry Products, 1998–2011. https://www.data.gov/.

[17] U.S. Department of Agriculture FSIS Risk Assessment for Guiding Public Health-Based Poultry Slaughter Inspection, 2012. https://www.data.gov/.

[18] Dookeran M.M., Baccus-Taylor G.S., Akingbala J.O., Tameru B., Lammerding A.M. (2012). Assessing thermal inactivation of salmonella on cooked broiler chicken carcasses in Trinidad and Tobago. Open Conf. Proc. J..

[19] Alali W.Q., Thakur S., Berghaus R.D., Martin M.P., Gebreyes W.A. (2010). Prevalence and distribution of Salmonella in organic and conventional broiler poultry farms. Foodborne Pathog. Dis..

[20] Maijala R.T., Ranta J., Seuna E., Pelkonen E., Johansson T. (2005). A Quantitative Risk Assessment of the Public Health Impact of the Finnish Salmonella Control Program for Broilers. Int. J. Food Microbiol..

[21] World Health Organization & Food and Agriculture Organization of the United Nations (2002). Risk Assessments of Salmonella in Eggs and Broiler Chickens.

[22] McCullough N.B., Eisele C.W. (1951). Experimental human salmonellosis. 1. Pathogenicity of strains of Salmonella meleagridis and Salmonella anatum obtained from spray-dried whole egg. J. Infect. Dis..

[23] Peter F.M.T., Fumiko K., Aamir F., Iain D.O., Ovidiu R., Norval J.C.S. (2010). Dose–response modeling of Salmonella using outbreak data. Int. J. Food Microbiol..

[24] Food and Agriculture Organization of the United Nations Statistics Division. www.faostat.fao.org.

[25] RISK A. Joint FAO/WHO Expert Consultation on Risk Assessment of Microbiological Hazards in Foods. http://citeseerx.ist.psu.edu/viewdoc/download?doi=10.1.1.110.5983&rep=rep1&type=pdf.

[26] Volkova V.V., Bailey R.H., Wills R.W. (2009). Salmonella in Broiler Litter and Properties of Soil at Farm Location. PLOS ONE.

[27] Wray C., Davies R.H., Evans S.J. (1999). Salmonella Infection in Poultry: The Production Environment. Poultry Meat Science.

[28] Harrison W.A., Griffith C.J., Tennant D., Peters A.C. (2001). Incidence of Campylobacter and Salmonella isolated from retail chicken and associated packaging in South Wales. Letters in applied microbiology. Lett. Appl. Microbiol..

[29] Gosia K.K., Hélène C., Thomas G., Kim H., Paulett M., Thuy P., Jeffrey M.F. (2010). Safe Endpoint Temperature for Cooking Whole Raw Poultry: Health Canada Recommendation. Food Prot. Trends.

[30] Vandeplas S., Dauphin R.D., Beckers Y., Thonart P., Thewis A. (2010). Salmonella in chicken: Current and developing strategies to reduce contamination at farm level. J. Food Prot..

[31] Lammerdinga A.M., Aamir F. (2000). Hazard identification and exposure assessment for microbial food safety risk assessment. Int. J. Food Microbiol..

